# Nervonic Acid Synthesis Substrates as Essential Components in Profiled Lipid Supplementation for More Effective Central Nervous System Regeneration

**DOI:** 10.3390/ijms25073792

**Published:** 2024-03-28

**Authors:** Magdalena Namiecinska, Paweł Piatek, Przemysław Lewkowicz

**Affiliations:** Department of Immunogenetics, Medical University of Lodz, Pomorska 251/A4 Street, 92-213 Lodz, Poland; pawel.piatek@umed.lodz.pl (P.P.); przemyslaw.lewkowicz@umed.lodz.pl (P.L.)

**Keywords:** nervonic acid, PUFAs, natural fish oil, neurodegenerative diseases, remyelination, CNS regeneration

## Abstract

Central nervous system (CNS) damage leads to severe neurological dysfunction as a result of neuronal cell death and axonal degeneration. As, in the mature CNS, neurons have little ability to regenerate their axons and reconstruct neural loss, demyelination is one of the hallmarks of neurological disorders such as multiple sclerosis (MS). Unfortunately, remyelination, as a regenerative process, is often insufficient to prevent axonal loss and improve neurological deficits after demyelination. Currently, there are still no effective therapeutic tools to restore neurological function, but interestingly, emerging studies prove the beneficial effects of lipid supplementation in a wide variety of pathological processes in the human body. In the future, available lipids with a proven beneficial effect on CNS regeneration could be included in supportive therapy, but this topic still requires further studies. Based on our and others’ research, we review the role of exogenous lipids, pointing to substrates that are crucial in the remyelination process but are omitted in available studies, justifying the properly profiled supply of lipids in the human diet as a supportive therapy during CNS regeneration.

## 1. Introduction

Central nervous system (CNS) degeneration, as the progressive, structural, and/or functional decline of neural tissue, can be caused by chronic inflammation, as in multiple sclerosis (MS) mediated by autoreactive lymphocytes or connected with the natural ageing process. However, both processes are associated with abnormal reconstruction and functioning of the myelin sheath [1].

The myelin, as a specialised multilamellar membrane consisting of 40 or more tightly wrapped lipid bilayers, which account for 70–75% of its dry weight, is complemented with an array of myelin-specific proteins [2,3]. Although it is characterised by a high lipid-to-protein ratio, among the proteins, the most essential are proteolipid protein (PLP), myelin basic protein (MBP), and myelin oligodendrocyte glycoprotein (MOG). The interactions between both lipids and proteins are crucial for myelin formation and function. Lipids control protein sorting, while myelin proteins are responsible for arranging lipids and creating regions of specialised molecular packing (e.g., lipid rafts) related to their functioning [4]. Therefore, it is so important that the synthesis of both components, stromal proteins and lipids, occurs simultaneously and spatiotemporally. The three well-known classes of membrane lipids are cholesterol, phospholipids (e.g., plasmalogen, lecithin, sphingomyelin), and glycolipids (e.g., galactosylceramide). In turn, the lipid composition of the myelin sheath is different, with high amounts of cholesterol and glycolipid in a ratio of 40%:40%:20% (cholesterol, phospholipid, and glycolipid, respectively) compared to other biological membranes (25%:65%:10%) [5]. In humans, myelin is produced within the first two post-natal years, while the myelination process continues until the third life decade [6]. Despite the lipid–protein ratio remaining stable throughout myelin development, marked changes are observed in the level of several lipid species, pointing to their functional modulation at different stages. The obtained data revealed marked changes observed in the amounts of cerebroside, sulfatide, and phosphatidylcholine (the total galactolipid increased from 21% of the total lipid at age 15 days to about 31 per cent at maturity, while phosphatidylcholine decreased from 17 to 11%) [7,8]. The appropriate lipid supplementation to obtain therapeutic benefits may contribute to effective CNS regeneration and repair as a supportive therapy. 

Disturbances in the proper reconstruction of myelin in the CNS are the bases of the pathology of MS, as chronic inflammation permanently accompanies new myelin synthesis. It has been suggested that oligodendrocytes (OLs) and their dysfunction in forming the myelin sheath of neurons are important factors underlying incomplete remyelination. The newly formed myelin sheaths in remyelinating lesions are thinner than those observed during development [9], and unmyelinated fibres are particularly vulnerable to further damage [10].

Until recently, it was assumed that the vast majority of myelinating OLs remained stable throughout life. Unfortunately, it has been confirmed that during the ageing process, the ability of oligodendrocyte precursor cells (OPCs) to differentiate decreases, which may cause dysfunction or a lack of OLs. This is just one of the causes underlying the aetiology of ageing-related demyelination, in addition to the loss of myelin proteins and age-associated changes in microglia and astrocytes that influence the maturation process of OPCs. Therefore, there is a decline in myelin plasticity with age, which results in limiting the efficiency of remyelination after injury [1].

All fatty acids can be taken up from the diet, and they are categorised into two main groups based on the capacity of the organism to synthesise them (non-essential fatty acids (FAs)), or not (essential fatty acids). These essential FAs are represented by alpha-linolenic acid (ALA, C18:3 n-3) and linoleic acid (LA, C18:2 n-6), because of the lack of enzymes (Δ12- and Δ15 desaturases) necessary for their synthesis in humans. Non-essential fatty acids can be synthesised by the organism or derived in the body from essential amino acids and include, among others, arachidic acid, stearic acid, and palmitic acid. The blood–brain barrier (BBB) is permeable to fatty acids, allowing the transport of both groups into the CNS, where astrocytes and oligodendrocytes can metabolise them [11]. 

The crucial role that lipids play in CNS development is highlighted by the high number of neurometabolic diseases caused by mutations in genes encoding enzymes, co-factors, or transporters involved in lipid metabolism [6,12]. Despite increasing attention being focused on fatty acids, most research concentrates only on LA and ALA as substrates for well-described eicosapentaenoic acid (EPA, 20:5 n-3) and docosahexaenoic acid (DHA, 22:6 n−3) with a crucial role in CNS regeneration [13]. However, too little attention is paid to defects in the biosynthesis of very-long-chain fatty acids, including nervonic acid (NA, 24:1 n-9), which accompanies impaired myelination. Important substrates for the synthesis of NA, requiring attention in appropriate supplementation, are palmitic acid, stearic acid, oleic acid, and eicosenoic acid, with the first three being a common link for the synthesis of both nervonic and arachidonic acid. Under physiological conditions, providing all of the mentioned substrates in the diet can result in increasing the synthesis of NA, while in the course of demyelinating diseases, providing palmitic acid, stearic acid, and oleic acid can also influence the alternative pathway of pro-inflammatory arachidonic acid synthesis [14]. In addition to an appropriate diet, an extremely important aspect is additional supplementation with fish oil, which is a source of all the necessary substrates mentioned above (Table 1).

Thus, this review aims to highlight the crucial role of properly selected substrates for NA synthesis as well as other components critical in CNS regeneration and the need to include them in the profiled lipid supply.

## 2. How Are the Lipid Components Necessary for Myelin Formation Supplied?

Fatty acids (FAs) are essential components of sphingomyelin, phosphatidylcholine, ceramide, glucosyl-ceramide, and sulfatide, constituting the membrane. As lipoprotein particles do not cross the blood–brain barrier (BBB) under normal circumstances, the lipids important for myelin synthesis must be produced de novo in the CNS, while their synthesis is greater in demyelinating diseases like MS [17]. The CNS expresses genes for FA synthesis and is autonomous in lipid metabolism [18]. The main one is the fatty acid synthase (FASN) gene, which encodes the multifunctional enzyme, FASN, crucial in de novo fatty acid synthesis, which catalyses the synthesis of palmitate from acetyl-CoA and malonyl-CoA. Interestingly, FASN deficiency was shown to affect the correct maturation of oligodendrocytes and myelination [19]. Additionally, fatty acid elongase 4 (ELOVL4), a member of a large family of fatty acid elongases (ELOs) that catalyses the rate-limiting step in the elongation of long-chain fatty acids (LC-FAs), is proven to play a role in the production of very-long-chain fatty acids (VLCFA) that form the myelin sheath. Its expression is mainly observed in neurons, although some ELOVL4-positive cells have also been detected in brain white matter, suggesting potential expression by oligodendrocytes. However, the role of ELOVL4 and VLC-SFAs in oligodendrocytes responsible for myelination has not been studied to date [20]. 

Depending on target substrates, ELOVL1, 3, and 6 elongate SFAs and MUFAs, whereas ELOVL2, 4, and 5 elongate PUFAs [21]. Equally important is stearoyl-CoA desaturase-1 (SCD1), a key enzyme in fatty acid metabolism, responsible for forming a double bond in stearoyl-CoA. SCD1 is important to generate NA, is essential for remyelination, and has been shown to control the inflammatory phenotype of myelin phagocytosing macrophages and microglia and thereby impact inflammatory and repair processes. SCD1 inhibition is proven to promote a more anti-inflammatory phenotype in microglia and macrophages, resulting in an enhanced remyelination process [22].

During physiological brain development, astrocytes deliver a fraction of the lipids incorporated into myelin. They produce lipid transporter apolipoprotein E (ApoE) and release cholesterol to LDL particles for uptake by OLs and incorporation into the myelin sheath [23]. ApoE is mainly expressed in astrocytes and microglia and appears in three major isoforms, ApoE2, ApoE3, and ApoE4. In astrocytes, ApoE4 impairs FA metabolism by enhancing de novo FA synthesis, reducing FA degradation, and promoting lipid droplet accumulation [24].

It has been proved that allele ε4 of the ApoE gene encoding the apolipoprotein E is a major genetic risk factor for Alzheimer’s disease (AD) [25]. Generally, lipid synthesis by astrocytes and horizontal lipid flux to oligodendrocytes for myelin synthesis or repair is a main feature of physiological brain development. Moreover, data show that dietary lipids compensate for diminished lipid synthesis in the brain [26]. As we have shown in our studies, the exogenous lipid intake influences the lipid profile in serum, justifying the use of appropriate diet [27]. Taking into account the presence of enzymes that form a double bond at 9C and lipid transporters, the organism is also prepared to synthesise more complex acids, such as nervonic acid from exogenous substrates.

We should draw knowledge about the body’s needs related to the proper development of the nervous system from “nature” itself, based on the composition of the mother’s milk, which is adapted to the development of the child’s brain. During the first year of a child’s life, the size of the brain increases almost threefold, and its development primarily concerns the growth of neurons; mother’s milk is prepared to provide appropriate and optimal nutrient intakes.

Both with age and in demyelinating diseases, the myelin sheath is gradually damaged, and its “renewal” is necessary, which means providing appropriate substrates and creating a favourable microenvironment for the remyelination process. Thus, the optimal supply of exogenous lipids for proper myelin sheath synthesis should be based on the composition of fatty acids in mother’s milk, which is like a “gold standard”. According to worldwide data, mother’s milk contains 42.2% saturated fatty acids (SFAs), 36.6% monounsaturated fatty acids (MUFAs), and 21.0% polyunsaturated fatty acids (PUFAs), whereas, among MUFAs, oleic acid (C18:1n-9) is the major component. The described lipid profile of human milk includes the concentrations of total fat; phospholipids; cholesterol; the SFAs 6:0, 8:0, 10:0, 12:0, 13:0, 14:0, 15:0, 16:0, 17:0, 18:0, 20:0, 22:0, and 24:0; the MUFAs 16:1n–7, 18:1n–9, 20:1n–9, and 22:1n–9; and the PUFAs as LA, 18:3n–6, 20:2n–6, 20:3n–6, 20:3n–3, 20:4n–6 (arachidonic acid (AA)), ALA, EPA, DHA, total n–6, and total n–3 [28]. Alterations in maternal diet with different intakes of PUFAs are reflected in the fatty acid composition in breast milk [29] if the mother has an extremely high intake of fish or is a vegetarian [30]. As a dietary supply of PUFA is essential for infant brain development and function, mother’s milk provides already-synthesised DHA and AA as well as their respective C18 precursors, LA and ALA, that can be converted to LCPUFA after ingestion. Although the brain can generate some DHA by endogenous synthesis from precursor n-3 LCPUFA in glial cells [31], DHA synthesis in the brain occurs at a much lower rate than the total rate of brain accretion of DHA needed to support growth and development [32]. In addition to DHA and EPA, which are well described in the literature, substrates for the synthesis of NA have been proven to be key components of sphingolipids, accounting for approximately 35% of all myelin lipids. Many recent studies indicate that NA is a crucial molecule for maintaining brain physiology as the main component of myelin [33,34]. Unlike other tissues, NA synthesis appears to be a critical process in the brain. Based on the biochemical pathway of its synthesis, we conclude that most mother’s milk components appear as substrates for the synthesis of NA and are important for the proper construction of myelin; therefore, they cannot be omitted during targeted lipid supplementation to support CNS development and regeneration.

## 3. Substrates for NA Synthesis Proved to Be Crucial in the Remyelination Process

In addition to well-described EPA and DHA, NA and substrates for its synthesis have recently attracted considerable attention because of their crucial role in demyelinating diseases [35]. It is worth adding that abnormal NA levels are tightly correlated with a high risk of developing neurological disorders or mental illnesses such as psychosis, schizophrenia, or attention deficit disorder [36,37], and supplementation with oils rich in EPA/DHA/NA reduces the clinical symptoms of the aforementioned diseases.

NA combines with sphingosines via an amide bond, forming nervonyl sphingolipids, an important component in the white matter of brains and myelinated nerve fibres. NA is synthesised from oleic acid (OA, C18:1 n-9) by carbon chain elongation, where two C units donated by malonyl-CoA are cyclically added to the acyl chain. Its production in the human body occurs due to conversion from other fatty acids through a series of biochemical reactions; another way is direct ingestion, by which NA is readily available to the body [38]. 

**Figure 1:**
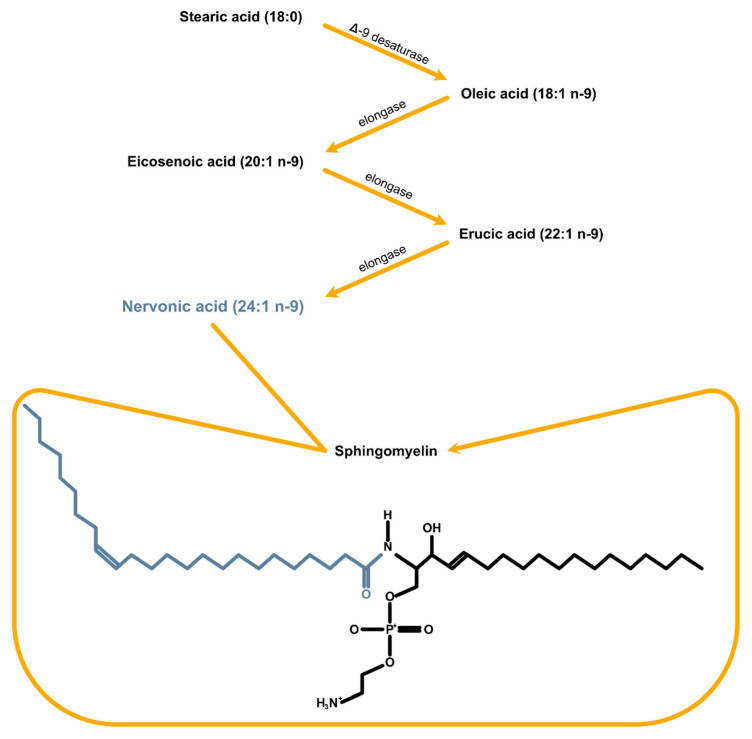
NA synthesis pathway and its incorporation into sphingomyelin.

The initiating step of NA synthesis is mediated by stearoyl-CoA (Δ9) desaturase (SCD). It converts saturated palmitic acid (C16:0) or stearic acid (C18:0) into their mono-unsaturated counterparts, palmitoleic acid (C16:l n-7) and oleic acid (C18:l n-9), respectively (Figure 1.) Oleic acid is a major FA component of myelin and a crucial factor for brain development, promoting neural cell differentiation and axonal growth [39]. Subsequently, FA elongase-1 lengthens oleic acid (C18:1 n-9) into erucic acid (C22:1 n-9), and finally, NA. It can be a marker of neurodegeneration, and its intake improves brain development [40,41]. Moreover, the latest publication confirms that NA also has beneficial effects on risk factors connected with adrenoleukodystrophy (ALD) [42], obesity and diabetes [43,44] or inflammatory bowel disease (IBD) [45], and understanding critical mechanisms should become a therapeutic approach [46]. It is noteworthy that the enzymes of n-3 fatty acid interconversion are shared with the analogous n-6 fatty acid biosynthetic pathway of conversion of LA to AA. Thus, the availability of substrates that compete with the same enzymes is considered to be crucial in directing FA to pro- or anti-inflammatory actions depending on the end product. This explains the support for high intakes of omega-3 rather than omega-6 FA.

Since substrates compete with enzymes common for the nervonic acid pathway and omega-3,-6 synthesis, an adequate intake of lipid substrates can affect the composition and function of the myelin sheath. The continuous regeneration of the myelin sheath and the accumulation of ‘inappropriate’ lipids in its composition can result in insufficient remyelination.

It has been shown that cells involved in the inflammatory response contain a high proportion of the n-6 fatty acid, AA, in their membrane phospholipids. Moreover, eicosanoids produced from AA play important roles in inflammation. Oral supplementation with n-3 fatty acids, EPA and DHA, increases their contents in the membranes of cells involved in inflammation with a simultaneous decrease in the amount of AA. Thus, different types of fatty acids can influence inflammatory cell function and modify inflammatory processes [47]. As n-3 PUFAs act oppositely to the pro-inflammatory n-6 PUFAs, their role in regulating inflammatory processes and responses suggests that the level of exposure to particular fatty acids is important in determining the development and severity of inflammatory diseases.

The incorporation of EPA and DHA into cell membranes is typically at the expense of n–6 fatty acids, including AA. This is crucial in supporting immune function and controlling inflammation. Some eicosanoid mediators produced from AA such as prostaglandin (PG) E2 have immunosuppressive effects, decreasing the function of T and B cells, while PGE2, PGD2, and several of the 4-series leukotrienes are involved in the inflammatory response [48].

Like DHA and EPA, NA becomes a strong candidate for further evaluation as a lipid supplement due to its excellent biofunctions in promoting human health, particularly CNS regeneration.

## 4. Effective Remyelination Based on the Properly Profiled Lipid Intake

We are grounded in dogma about the miraculous effects of oils, observing, for example, their anti-inflammatory effects. The therapeutic properties of shark liver oil were described already in the 1970s, when alkylglycerols, with no side effects, were introduced as an immune booster in infectious diseases [49]. Oils of certain plants and nuts, such as flax seed oil, are considered rich dietary sources of ALA, while fatty fish (e.g., salmon and mackerel) are rich dietary sources of EPA, DPA, and DHA [50]. Nowadays, we can fully use their properties by dedicating them to the appropriate disease entity. We now know that fish oil (FO) is the main source of omega-3 PUFAs and is widely described in the literature to have beneficial effects on the proper functioning of the brain and cardiovascular system or the eye retina. Recent studies concentrated on the beneficial effects of the intake of n-3 PUFAs in a variety of neurodegenerative and neurological diseases due to their neuroprotective and anti-inflammatory properties [51]. Unfortunately, most literature reports focus only on maintaining appropriate DHA levels in neural cell membranes as DHA is essential for their functions and has been proven to prevent neuronal damage or apoptosis [52,53]. EPA and DHA have been shown to cross the BBB by simple diffusion and mediate neuroprotection via prolonging the lifespan of glial cells and inhibiting microglia and inflammatory cells [54]. These are just a few examples of the beneficial effect of omega-3 on the regeneration of the CNS, which are described in detail in many reviews [51,55]. However, no one has conducted research on isolated EPA and DHA fractions; they have only in oil mixtures, which has meant the influence of other ingredients that occur in negligible amounts has been ignored, but as it turns out, they perform no less important functions. Based on our research, this review sheds new light on the need for appropriate profile lipid supplementation, including substrates for NA synthesis, to achieve a more effective influence on CNS regeneration, including the remyelination process. 

In spite of the similar content of EPA and DHA, in our studies, with the use of the RP-LC-Q-Orbitrap HRMS technique, we have proved that an FO mixture contains significantly more lipid species than borage oil (BO) (335 FOM vs. 204 BO). We have shown the spectacular differences in species number, mainly concerning triacylglycerols (TGs) (232 FOM vs. 151 BO) and diacylglycerols (DGs) (78 FOM vs. 48 BO), without significant differences in the percentage of TG (94.7% FOM vs. 93.7% BO) and DG (5.3% FOM vs. 6.3% BO). Despite the similar composition of both oils in terms of main compounds, FOM has been proven to possess multidirectional potential affecting myelin protein synthesis, the NA pathway, and growth factors compared to BO [56]. This indicates an extremely important but still underestimated role of compounds present in small amounts, which influence the activation of the process of the synthesis of myelin proteins and growth factors via the receptor pathway.

Remyelination, which follows the pathological loss of myelin, is the phenomenon by which new myelin sheaths are generated around axons in the adult CNS. It was shown that the sphingomyelin, cerebroside, and sulfatide fractions from MS myelin were characterised by the increased level of palmitic and stearic acids and decreased NA compared to control myelin [57]. Significantly decreased sphingomyelin and increased phospholipids were found in MS myelin [58]. Moreover, in MS white matter, C16:0- and C18:0-ceramide increases were correlated with oligodendrocyte apoptosis [59]. In our studies, the whole brain profiling of lipid composition in an experimental autoimmune encephalomyelitis (EAE) animal model of MS revealed the dysregulation of and decrease in NA synthesis, as well as the increased synthesis of AA. As both NA and AA synthesis used the same substrates, the switch in the lipid synthesis pathway might be mediated by an inflammatory environment in the brain [14]. Since OLs are cells responsible for producing vast amounts of lipids and proteins during the formation of the myelin sheath, ineffective remyelination may be related to the lack of essential lipid substrates provided in the adult human diet [60].

In our research, we have checked the effect of a fish oil (FO) mixture (patent No. UPRP P.416768) rich in NA and n-3 PUFAs on a human model of maturating OPCs. In our in vitro model of human oligodendrocyte precursor cell (hOPC) maturation, the main source of NA synthesis came from the palmitic (C16:0)/stearic (C18:0)/oleic (C18:1n9c) acid ester pathway. Moreover, linoleic acid ester (C18:2n6c) was a product catalysed by ∆6 desaturase for transformation into oleic acid (C18:1n9). Moreover, there is a direct possibility for DHA to transform into NA [14]. Concentrating on studies with naturally occurring NA, we revealed that the physiological synthesis of both myelin components, proteins and lipids, was significantly enhanced after BBB supplementation with FO. We noted that hOPCs in the environment created by BBB cells increased the synthesis of myelin proteins MBP, MOG, and PLP about twice and additionally polarised lipid metabolism on the NA pathway synthesis [56]. In the direct incubation of astrocytes and ECs with a fish oil mixture, we observed the increase in NA lipid products (oleic acid, cis-11-eicosenoic acid, erucic acid, and nervonic acid) in these cells. Our research on lipid profiling in an experimental autoimmune encephalomyelitis (EAE) brain revealed that the silenced synthesis of NA in acute inflammation is the result of shifting the lipid metabolism pathway of common substrates into proinflammatory AA production. The intake of oil mixtures rich in NA esters affects OPC maturation, improves OL function, and thus increases the effectiveness of remyelination [56]. Additionally, we proved the positive influence of FO mixture supplementation on growth factors released during the polarisation of hOPCs to mature OLs. It resulted in an increased concentration of IGF-1 (insulin-like growth factor 1), BDNF (brain-derived neurotrophic factor), and CNTF (ciliary neurotrophic factor), relevant factors possessing neurodegenerative effects. Moreover, based on the literature and our obtained data, we postulated that the activation of FFAR1 and FFAR4 receptors by long-free fatty acids is an essential step to initiate the synthesis of both proteins and lipids forming the myelin sheath. In turn, recent studies by the Zhou group have confirmed that enhanced lipid metabolism by the activation of the linoleic acid pathway restores microglial pro-regenerative properties, resulting in better recovery from demyelination injuries [61]. Moreover, another study, including precursors for NA synthesis, proved that early dietary treatment with Lorenzo’s oil (containing EA: oleic acid; 4:1) and DHA has a positive influence on neurological development in patients with Zellweger syndrome [62] and ALD, where demyelination is associated with an accumulation of very-long-chain saturated fatty acids such as C26:0. It was proved that erucic acid and NA can normalise elevated serum levels of C26:0 in ALD [63].

Generally, risk factors contributing to neurodegenerative diseases have been described by the gut–brain axis hypothesis that includes stress, an unbalanced diet, and drug impacts altering microbiota composition [64]. It has been shown that proper diet can control MS disease and reduce its progression [65]. Consuming more PUFAs, especially fish oil and n-3PUFAs, has been shown to reduce the progression of MS. The dietary intake of n-3 PUFAs (mainly EPA and DHA) decreased the production of tumour necrosis factor-alpha (TNF-α), interleukin-1 beta (IL-1β), and interleukin-6 (IL-6), simultaneously increasing the level of the anti-inflammatory cytokine interleukin-10 (IL-10) [66,67]. In our studies, we have postulated the need to include nervonic acid esters in lipid supplementation to achieve better results in CNS regeneration. Our research with a properly selected FO mixture rich in nervonic acid substrates pointed to the inhibition of the secretion of several proinflammatory factors by human OPCs. They can be involved in immune cell recruitment during the remyelination process and in promoting growth factor synthesis, important for CNS regeneration. Our findings point out that the intake of an FO mixture rich in the nervonic acid ester may improve OL function, affecting OPC maturation and improving myelin synthesis, and thereby the remyelination process [14]. 

Clinical trials are mainly targeted at n-3 PUFAs, especially EPA and DHA, and their beneficial effects have been related to their neuroprotective and anti-inflammatory properties in various neurodegenerative diseases and neurological diseases [68], as well as many being connected with depression, migraine, ADHD (attention-deficit/hyperactivity disorder), and dementia. The clinical studies available in the database (https://clinicaltrials.gov/, search path: ‘omega 3, CNS’, ‘omega 6, CNS’, omega 9, CNS’ accessed of 21 March 2024) regarding the positive impact of omega-3,-6,-9 on the CNS are as follows: omega-3 (146 trails; 90 completed), omega-6 (51 trials, 28 completed). It should be noted that some studies cover both omega-3 and -6 (Table 2). Although clinical trials are taking into account omega-9, they are concerned about its effect only in connection with omega-3 and 6, with no beneficial effect of omega-9 itself on CNS renewal and remyelination being discussed. The lack of clinical studies on the positive aspects of omega-9 shows that despite its proven effect on the CNS and remyelination process, its effect is still underestimated and requires further research.

Moreover, our studies prove the wide spectrum of lipid activity in other diseases, as dietary supplementation with a high dose of omega-3 PUFA during non-surgical therapy also has potential benefits in periodontitis [69]. Most of the published epidemiological studies are consistent with a positive correlation between high DHA consumption and a lower risk of developing AD later in life [70]. Thus, recent studies provide us with an opportunity to use PUFAs as a pharmaceutical tool in many diseases, particularly brain disorders such as Alzheimer’s disease (AD) and Parkinson’s disease (PD) for the prevention of the development of these diseases in elderly people and people bearing a genetic risk for AD.

Taking into account the lipid composition of the myelin sheath and mother’s milk, the role of substrates of the NA synthesis pathway in the process of CNS regeneration cannot be ignored and should be treated in parallel with conventional treatment as an essential element of successful recovery or slowing down the CNS ageing process. There are many indications that the documented regenerative effect of EPA and DHA can be contributed by NA and the substrates of its synthesis pathway, which, however, are omitted in the recommended supplementation. 

## 5. Conclusions and Future Perspectives

Despite solid evidence of the crucial role of NA substrates in CNS regeneration, many questions still remain open. Taking into consideration their documented regenerative effect, we strongly postulate to include them in profiled lipid supplementation for more effective remyelination, as myelin lipid homeostasis is critical for long-term myelin stability. Since most of the research focuses mainly on omega-3 and omega-6 fatty acids, it is reasonable to conduct future clinical trials taking into account substrates for NA synthesis. In the light of ongoing research, available lipids with a proven beneficial effect on the remyelination process could be included as a supportive therapy in MS. Moreover, the regenerative effect of fatty acids, including the underestimated omega-9, can be used in other neurodegenerative diseases, such as Alzheimer’s and Parkinson’s disease or amyotrophic lateral sclerosis. Current studies show that since inflammation and lipid dysregulation have been associated with AD pathology, the combination of proper lipids together with fish oil has the potential to maximise therapeutic benefit by acting on all mechanisms associated with disease pathology. Appropriate supplementation with fish oil may also reduce the risk of developing dementia. In conclusion, lipid involvement in CNS renewal is still a largely unexplored field but can lead to the discovery of critical functions for a different subset of lipid classes mediating myelin development. There is increasing evidence that fish oils with appropriate proportions of selected fatty acids can be the recommended supplementation to the daily diet that cannot provide all essential lipid components for more effective CNS regeneration.

## Figures and Tables

**Table 1:** Dietary sources of NA synthesis substrates [15,16].

NA Synthesis Substrates	Sources
Palmitic acid (16:0)	Palm oil, meat, poultry, olive oil, dairy products, soyabean oil, eggs, fish oil
Stearic acid (18:0)	Meat (beef), poultry, soyabean oil, olive oil, butter, sunflower oil, fish oil
Oleic acid (18:1 n-9)	Olive oil, soyabean oil, nuts, sesame oil, sunflower oil, fish oil
Eicosenoic acid (20:1 n-9)	Nuts, fish, plant oils, fish oil

**Table 2:** Clinical trials with the positive impact of omega-3 and -6 fatty acids.

	Clinical Trial	Treatment Group	Placebo Group	Duration (Months)	Purpose	Clinical Trial ID
Omega 3/omega 6	Lipoic Acid and Omega-3 Fatty Acids for Alzheimer’s Disease	34 + lipoic acid and fish oil concentrate	33 + placebo lipoic acid plus placebo oil	18	To see if taking lipoic acid plus omega-3 fatty acids (omega-3s) can slow the Alzheimer’s disease (AD) process.	NCT01058941
	DHA (Docosahexaenoic Acid), an Omega 3 Fatty Acid, in Slowing the Progression of Alzheimer’s Disease (DHA)	238 + DHA	164 + placebo	18	To determine whether chronic DHA (docosahexaenoic acid) supplementation slows the progression of cognitive and functional decline in mild to moderate Alzheimer’s disease (AD).	NCT00440050
	Fish Oil and Alpha Lipoic Acid in Treating Alzheimer’s Disease	13 + fish oil concentrate	13 + placebo oil (soybean oil)	12	To evaluate the effect of fish oil and alpha lipoic acid on inflammation, lipid levels, and oxidative stress.	NCT00090402
	Fish Oil for the Treatment of Depression in Patients with Multiple Sclerosis	21 + ish oil concentrate	18 + soybean placebo	3	To determine whether fish oil can reduce depression in people with multiple sclerosis (MS).	NCT00122954
	Efficacy of Fish Oil in Multiple Sclerosis (EFOMS)	50 (RR-MS) + fish oil	No data	12	To evaluate the efficacy of fish oil supplementation on serum proinflammatory cytokines levels, oxidative stress markers, and disease progression in MS.	NCT01842191
